# Probing myocardial blood oxygenation reserve with controlled hypercapnia using BOLD CMR

**DOI:** 10.1186/1532-429X-16-S1-O14

**Published:** 2014-01-16

**Authors:** Hsin-Jung Yang, Roya Yumul, Richard Tang, Ivan Cokic, Michael Klein, Avinash Kali, Olivia Sobczyk, Behzad Sharif, Jun Tang, Xiaoming Bi, Sotirios A Tsaftaris, Debiao Li, James K Min, Daniel S Berman, Antionio Hernandez Conte, Joseph A Fisher, Rohan Dharmakumar

**Affiliations:** 1 Cedars Sinai Medical Center, Los Angeles, California, USA; 2 Bioengineering, University of California, Los Angeles, Los Angeles, California, USA; 3 Anesthesiology, University of California, Los Angeles, Los Angeles, California, USA; 4 Physiology, University of Toronto, Toronto, Ontario, Canada; 5 Anesthesiology, University of Toronto, Toronto, Ontario, Canada; 6 IMT institute for advanced studies lucca, Lucca, Italy; 7 Siemens Medical Solusions, Chicago, Illinois, USA

## Background

Background More than half of the cardiac stress tests require pharmacologic vasodilators for induction of hyperemia to assess myocardial perfusion, but carry the potential for side effects and are contraindicated in many patients considered for testing. We evaluated the feasibility of a non-invasive and safe stress-testing paradigm using a precisely targeted partial pressure of arterial CO2 (PaCO2) to induce myocardial hyperemia, and compared this response to intravenous adenosine.

## Methods

Dose-response studies were performed on spontaneously breathing humans (n = 18), and canines (n = 18) with and without surgically implemented coronary stenosis to determine the optimal increase in PaCO2 required to replicate the hyperemic response to intravenous adenosine (140 μg/kg/min). Blood-Oxygen-Level-Dependent (BOLD) CMR was used to determine the effects of hypercapnea.

## Results

In humans, an increase in PaCO2 of 10 mmHg was well tolerated, and the BOLD CMR responses were similar to those due to standard adenosine (p = 0.7). In intact canines, the BOLD response to a mean increase in PaCO2 of 11 mmHg was similar to that of adenosine infusion (140 μg/kg/min, p = 0.4); the responses were also similar in the territories subtended by stenotic (p = 0.7) vessels.

## Conclusions

Conclusion Targeted increases in PaCO2 of 10 mmHg is well tolerated and has a myocardial vasodilating effect similar in extent to that of adenosine. These findings support continued investigation into the feasibility of inhaled CO2 as a vasodilator for cardiac stress testing.

## Funding

National Heart, Lung, And Blood Institute (HL091989)

**Figure 1:**
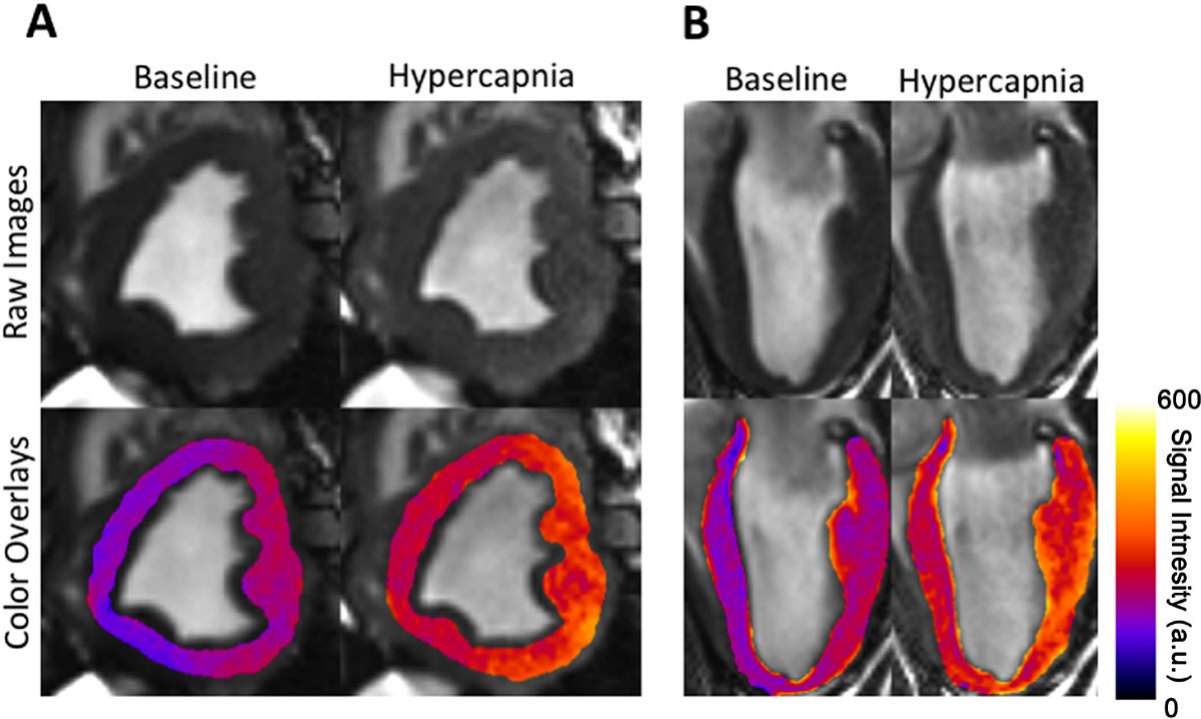
**Effect of changing arterial CO2 on BOLD CMR signal intensities**. Representative short (A) and long (B) axis BOLD CMR images collected from a canine from Group Ramp under baseline (PETCO2 = 42 mmHg) and hypercapnia (PETCO2 = 55 mmHg) are shown. Note the increase in signal intensity in images under hypercapnia relative to baseline.

**Figure 2:**
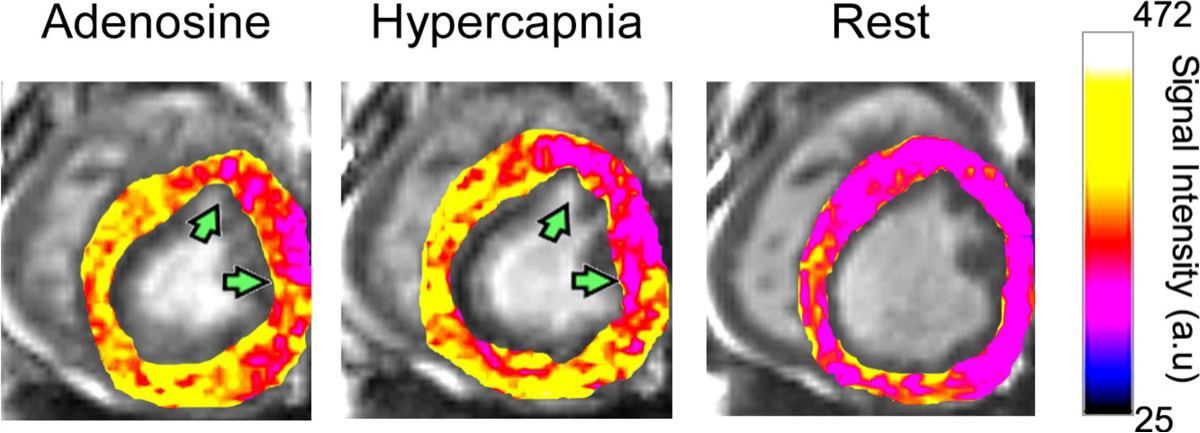
**BOLD CMR-based evaluation of adenosine versus hypercapnia in the presence of LAD stenosis in canines**. Representative color-overlaid BOLD CMR images acquired during adenosine infusion, hypercapnia, and rest from a canine with a significant narrowing of LAD. Color bar shows BOLD CMR signal intensity. Images acquired under adenosine infusion and at rest were obtained under normocapnia.

